# LncRNA KCNQ1OT1 enhanced the methotrexate resistance of colorectal cancer cells by regulating miR‐760/*PPP1R1B* via the cAMP signalling pathway

**DOI:** 10.1111/jcmm.14071

**Published:** 2019-04-17

**Authors:** Di Xian, Yu Zhao

**Affiliations:** ^1^ Department of Emergency Surgery Sichuan Academy of Medical Sciences & Sichaun Provincial People's Hospital Chengdu Sichuan China

**Keywords:** bioinformatics analysis, cAMP signalling pathway, colorectal cancer, lncRNA, methotrexate

## Abstract

We aimed to explore the mechanism of the KCNQ1OT1/miR‐760/*PPP1R1B* axis acting to regulate methotrexate (MTX) resistance of colorectal cancer (CRC). Differentially expressed mRNAs and lncRNAs in MTX‐sensitive CRC cell lines and MTX‐resistant cell lines were determined through microarray analysis. Application of bioinformatics analysis was aimed to uncover the relationships among the lncRNAs/miRNAs/mRNAs, and to demonstrate the effects of cAMP signalling pathway in MTX‐resistant CRC. The expression level of RNA and proteins was, respectively, detected using qRT‐PCR and Western blot assays, whereas the dual‐luciferase reporter gene assay was implemented to verify the targeted relationship. The influence of the lncRNA/miRNA/mRNA axis on biological functions of MTX‐resistant cells and on the growth of tumours determined through both vitro and vivo experiments. LncRNA KCNQ1OT1 and *PPP1R1B* mRNA were overexpressed in MTX‐resistant CRC tumour cells. KCNQ1OT1 functioned as a sponge of miR‐760, which targeted *PPP1R1B*. Knockdown of KCNQ1OT1 enhanced chemosensitivity towards MTX through the sponging of miR‐760. MiR‐760 expressed at low levels targeted *PPP1R1B* in the activated cAMP signalling pathway under MTX treatment. Knockdown of KCNQ1OT1 dampened the proliferation of MTX‐resistant (HT29/MTX) cells by regulating the miR‐760/*PPP1R1B* axis, which also induced cell cycle arrest together with apoptosis. KCNQ1OT1 regulated the expression of *PPP1R1B* and the downstream genes *CREB* and *CBP* in the cAMP signalling pathway. MTX showed a suppressive function on CRC progression. KCNQ1OT1 enhanced the MTX resistance of CRC cells by regulating miR‐760‐mediated *PPP1R1B* expression via the cAMP signalling pathway.

## 1 INTRODUCTION

Known as the fourth most lethal cancer, colorectal cancer (CRC) is simultaneously the third most common cancer in the worldwide.^1^ Although the survival rate of CRC patients after surgery or chemoradiotherapy has recently been increasing,^2^ resistance to drug therapy or radiotherapy in CRC has been found in recent studies.^3^, ^4^ The occurrence, development, and variation in CRC are regulated by a complex structure of oncogene and gene pathways.^5^ Unfortunately, there is insufficient information on the specific mechanism of the regulation process. To improve therapies for CRC and unclose the underlying molecular mechanism of drug resistance in CRC is necessary.^6^

As one of the earliest developed cytotoxic drugs, methotrexate (MTX) is a chemotherapy agent and an immune system suppressant which functions as an antimetabolite and antifolate therapy for treatment of various human cancers. However, resistance to MTX brings challenges to the clinical application in cancers. Therefore, determining the mechanism of MTX resistance in cancers is extremely urgent in the further investigations and beneficial to studies on how to overcome drug resistance to assure the efficacy of MTX therapies.^7^

Long noncoding RNAs (lncRNAs) are considered noncoding RNAs whose length extends beyond 200 nucleotides, and they lack protein‐coding functions.^4^ Increasing evidence indicates that lncRNAs are deeply involved in human cancers through the regulation of diverse cellular functions and biological processes including epigenetic modification and mRNA stabilization.^8^ LncRNAs also function as competing endogenous RNAs (ceRNAs) that sponge microRNAs (miRNAs), by which they influence the expression of target messenger RNA (mRNA).^2^ Chemoresistance to anticarcinogens that is associated with this process has been found in abnormal cancer tissues.^9^ KCNQ1OT1, an lncRNA connected with tumorigenesis and the development of cancers such as Wilms’ tumour and hepatocellular carcinoma,^10^, ^11^ was observed to be highly expressed in human CRC cells in a recent report.^12^ However, the detailed mechanism by which KCNQ1OT1 regulates drug resistance in CRC is still unclear.

MicroRNAs are classified as highly conserved single‐stranded noncoding RNA molecules that down‐regulate gene expression by establishing sequence‐specific linkages with the 3′ untranslated regions (3′UTRs) of homologous mRNA targets.^13^ MiRNAs are always aberrantly modulated in tumorigenesis and malignant transformation, acting as a part of the pathogenesis of cancer together with lncRNAs, as mentioned above.^14^ In terms of CRC cells, some altered miRNAs have been verified to control proliferation, metastasis, apoptosis and chromosomal instability.^13^, ^15^ In particular, miR‐760 affects the proliferation and invasion of CRC cells by involving in corresponding signalling pathways.^16^ However, its precise mechanism is still uncovered.

*PPP1R1B* is a gene which encodes protein phosphatase 1 regulatory subunit 1B. It performs a crucial role in brain functions. Additionally, the downstream proteins of *PPP1R1B* have been reported to be overexpressed in numerous cancers, such as oesophageal, gastric, colon, prostate, and breast cancers.^17^ This gene has an alias called *DARPP32*, which is a dopamine and cyclic AMP‐regulated phosphoprotein.^18^ The phosphorylation or dephosphorylation of *DARPP32* induced by cAMP signalling is still disputed. Furthermore, former studies on *PPP1R1B* have focused more on neurological and psychiatric disorders than on cancer, especially CRC, offering us a novel research direction.

Based on previous studies and our assumptions, the present experiments were devised to explore the regulatory mechanism of the KCNQ1OT1/miR‐760/*PPP1R1B* axis in MTX‐resistant CRC via the cAMP signalling pathway. This exploration may contribute to the discovery of new therapeutic targets and prognostic factors for CRC in the future.

## 2 MATERIALS AND METHODS

### 2.1 Microarray analysis

LncRNA and mRNA expression profiles from 3 pairs of MTX‐sensitive and MTX resistant CRC cells were selected from the Gene Expression Omnibus database (https://www.ncbi.nlm.nih.gov/geo/) (Series Accession Number GSE16066; Platform GPL570). Differentially expressed mRNAs and lncRNAs were sifted according to a threshold of | log_2_ (fold change) | > 1 and adjusted *P*‐value <0.05. Analyses was performed with R version 3.4.3 (https://www.r-project.org/) and the program package “Limma,” and the results are presented in a heatmap (green for RNAs with low expression and red for RNAs with high expression).

### 2.2 Coexpression network for lncRNAs and mRNAs

A coexpression network for differentially expressed mRNAs and lncRNAs was built using Cytoscape version 3.5.1 (http://www.cytoscape.org/). Node and edge files were produced by R according to the thresholds of Pearson coefficient factor >0.7 and *P* < 0.05. The solid line indicates a positive correlation between lncRNA and mRNA, whereas a dotted line indicates a negative correlation.

### 2.3 Gene set enrichment analysis

Gene set enrichment analysis (GSEA) was set about using GSEA software version 3.0. Significantly enriched signalling pathways were analysed based on the differentially expressed mRNAs and on data from the Kyoto Encyclopedia of Genes and Genomes database (threshold *P* < 0.05). Dot plots and ridge plots of the enriched pathways were generated using R with the “ggplot2,” “easygplot2,” and “clusterProfiler” program packages.

### 2.4 Tissue specimens

Colorectal cancer tumour tissues (n = 20) and MTX‐resistant CRC tumour tissues (n = 20) were collected from 40 CRC patients during the period between March 2016 and June 2017 at the Sichuan Academy of Medical Sciences & Sichaun Provincial People's Hospital of TCM. None of the patients received chemotherapy before sampling, and 20 of the samples presented strong resistance to MTX treatment. All resected tissue specimens were immersed in liquid nitrogen at once and maintained in a freezer at −80°C. This research had obtained the approval of by the Ethics Committee of Sichuan Academy of Medical Sciences & Sichaun Provincial People's Hospital of TCM, and written informed consent was acquired from all the patients.

### 2.5 Cell culture and MTX treatment

The human CRC cell line HT29 and Caco2 cells were purchased from BeNa Culture Collection Biological Technology Co., Ltd. (Beijing, China). The HT29 cells were cultured in RPMI‐1640 medium (Gibco, Grand Island, NY, USA) added with 10% foetal bovine serum (FBS; Gibco). The Caco2 cells were maintained in ATCC‐formulated Eagle's minimum essential medium supplemented with 20% FBS. All the cells were maintained in a humidified atmosphere of 5% CO_2_ incubator at 37°C. MTX‐resistant HT29 and Caco2 cells were obtained through incubation with stepwise concentrations of MTX (Sigma‐Aldrich, St. Louis, MO, USA). Resistant cells were routinely grown in selective DHFR medium lacking glycine, hypoxanthine and thymidine (‐GHT medium; Gibco) supplemented with 8% dialysed FBS. HT29 cells and Caco2 cells that were able to stably grow in 10 μmol/L MTX were selected as MTX‐resistant cells and labelled HT29/MTX and Caco2/MTX.

### 2.6 Cell transfection

Cells were cultivated in 6‐well plates (2 × 10^5^ cells per well). PcDNA 3.1‐KCNQ1OT1 plasmids, pcDNA 3.1‐*PPP1R1B* plasmids, miR‐760 mimics, a miR‐760 inhibitor, KCNQ1OT1 siRNA, *PPP1R1B* siRNA and pcDNA3.1 vector plasmids (NC) were all acquired from GenePharma (Shanghai, China). The above compounds were separately transfected into cells using Lipofectamine 3000 reagent (Invitrogen, Carlsbad, CA, USA) and Opti‐MEM serum‐free medium (Invitrogen) according to the manufacturer's protocol.

### 2.7 qRT‐PCR

Total RNA was harvested from cells utilizing TRIzol reagent (Invitrogen). The extracted RNA was reverse transcribed into cDNA with a PrimeScript™ RT Reagent Kit (Takara), and gene amplification using qRT‐PCR was performed following the instructions of the SYBR Premix Ex Taq™ GC master mix (Takara) on an ABI 7500 real‐time PCR system (Applied Biosystems). The relative gene expression was computed with the 2^−∆∆CT^ method, and GAPDH was selected as the internal control. The primer sequences used in qRT‐PCR are presented in Table 1.

**Table 1:** Primer sequences used in qRT‐PCR

Primers	Sequences
KCNQ1OT1 forward	5′‐TGCAGAAGACAGGACACTGG‐3′
KCNQ1OT1 reverse	5′‐CTTTGGTGGGAAAGGACAGA‐3′
miR‐760 forward	5′‐GTCGAGCGGCTCTGGGTCTGTG‐3′
PPP1R1B forward	5′‐GGCTGAAGTCCTGAAGGTCA‐3′
PPP1R1B reverse	5′‐CCCAGGTTCTCTGGGTATCA‐3′
GAPDH forward	5′‐TGCACCACCAACTGCTTAGC‐3′
GAPDH reverse	5′‐GGCATGCACTGTGGTCATGAG‐3′
U6 forward	5′‐TACGAGTGCTCACTFCGGCAGC‐3′
U6 reverse	5′‐GTCCTTGGTGcCCGAGTG‐3′

### 2.8 Dual‐luciferase reporter gene assay

Luciferase reporter vectors were constructed by chemically synthesizing the sequences of the predicted binding sites between wild‐type KCNQ1OT1 and miR‐760 and between wild‐type *PPP1R1B* and miR‐760 and inserting them into pmirGLO plasmids to interfere with luciferase gene expression (Promega). These reporter plasmids were named pmirGLO‐KCNQ1OT1‐wt and pmirGLO‐*PPP1R1B‐*wt. Similarly, the mutated binding sites were also synthesized and inserted into the reporter plasmid; these recombinant plasmids were named pmirGLO‐KCNQ1OT1‐mut and pmirGLO‐*PPP1R1B*‐mut. HT29/MTX cells and Caco2/MTX cells were cotransfected with the recombinant reporter plasmids and miR‐760 or negative controls using Lipofectamine 3000. After transfection and culturing in medium containing 10 μmol/L MTX for 2 days, the luciferase activity was probed with a Dual‐Luciferase Reporter Assay System (Promega). Firefly luciferase activity was standardized to that of Renilla luciferase.

### 2.9 Western blot

Radio immunoprecipitation assay lysis buffer (Sigma‐Aldrich) was employed for protein extraction. Then, the proteins were separated by SDS‐PAGE for 2 hours and were transferred onto a PVDF membrane, which was then soaked in TBST containing 5% skim milk at 37°C for 1 hour. The primary antibodies rabbit anti‐PPP1R1B (#ab40801, 1:1000; Abcam, Shanghai, China), rabbit anti‐CREB (#ab32515, 1:1000; Abcam), rabbit anti‐CBP (#ab2832, 1 μg/mL; Abcam), and rabbit anti‐β‐Actin (#ab8227, 1:1000; Abcam) were added to the membranes, and the membranes were then incubated at 4°C overnight. After being using TBST to wash three times the membranes were subsequently incubated for 2 hours at 37°C, with goat anti‐rabbit IgG H&L secondary antibody (1:2000). The immunological reaction was detected with ECL Plus reagent (Amersham, Piscataway, NJ, USA). ImageJ 1.8.0 software was conducted to analyse data. β‐actin was employed as an internal reference protein.

### 2.10 CCK‐8 assay

Approximately 1 × 10^5^ transfected cells per well in each group were seeded into a 96‐well plate, and then different concentrations of MTX (2.5, 5, 7.5, and 10 μmol/L) were added into separate wells. Following 72 hours of incubation, cell viability was testified using a Cell Counting Kit‐8 (CCK‐8; Dojindo, Kumamoto, Japan) under the manufacturer's instructions. The absorbance was read at 450 nm using a multifunctional microplate reader.

### 2.11 Flow cytometry analysis of cell cycle and apoptosis

Transfected cells were fixed with 70% cold ethanol for 3 hours, and then 100 μL of RNase was added. Following cultivation for 4 hours at 37°C, the cells were washed thrice with PBS after centrifugal separation. Subsequently, 0.5 mg/mL propidium iodide and 50 mg/mL RNase A were injected into the cells, and the cells were then subjected to fluorescence activated cell sorting (FACSCalibur; Becton Dickinson, Franklin Lakes, NJ, USA). FACS Diva (Becton Dickinson) software was used for flow cytometry data analysis.

### 2.12 Tumour xenograft

A total of 20 BALB/c nude mice were acquired from the experimental animal centre of Sichuan Academy of Medical Sciences & Sichaun Provincial People's Hospital of TCM and were divided into four groups: the HT29 group, the HT29/MTX group, the HT29/MTX+KCNQ1OT1 group, and the HT29/MTX+sh‐KCNQ1OT1 group. Tumour cells (1 × 10^7^) were injected into the right axillary fossa of every mouse, and MTX treatment started the second week after the tumour cell inoculation. MTX was administered via intraperitoneal injection twice every week. The tumour sizes were measured three times each week. The tumour volumes (*V*) were calculated based on the width (*w*) and length (*l*) according to the following formula: *V* = *l* × *w* ^2^/2. The mice were killed, and the tumour masses were determined after the 6th week. This animal experiment was authorized by the Ethics Committee of the Sichuan Academy of Medical Sciences & Sichuan Provincial People's Hospital of TCM and was performed following the institutional protocols.

### 2.13 Statistical analysis

The experiments mentioned above were repeated at least three times. GraphPad Prism 6.0 software (GraphPad Software, La Jolla, CA, USA) was used for data analysis. The data presented are the mean values (± SD) of three independent experiments. Differences between two groups were evaluated though Student's *t* test, whereas differences among multiple groups were calculated using ANOVA. *P* < 0.05 was deemed statistically significant.

## 3 RESULTS

### 3.1 Differentially expressed lncRNAs, mRNAs, and significantly altered pathways in MTX‐resistant CRC cells

**Figure 1:**
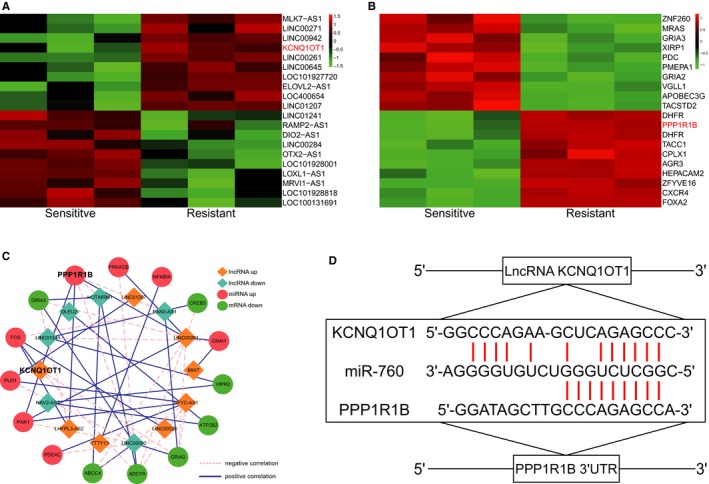
Differentially expressed lncRNAs and mRNAs in MTX‐resistant/sensitive CRC cells (A) The top 20 up‐ and down‐regulated lncRNAs were filtrated using microarray analysis. LncRNA KCNQ1OT1 was overexpressed in MTX‐resistant CRC cells compared with its expression in MTX‐sensitive cells, as shown in the heatmap. (B) The top 20 up‐ and down‐regulated mRNAs were selected through microarray analysis. *PPP1R1B* was up‐regulated in MTX‐resistant CRC cells compared with that in MTX‐sensitive cells, as shown in the heatmap. (C) Coexpression network of differentially expressed lncRNAs and mRNAs. KCNQ1OT1 was found to be correlated with *PPP1R1B*. (D) The binding sites of lncRNA KCNQ1OT1, *PPP1R1B,* and the specific miRNA (miR‐760) were determined with TargetScan and miRanda

**Figure 2:**
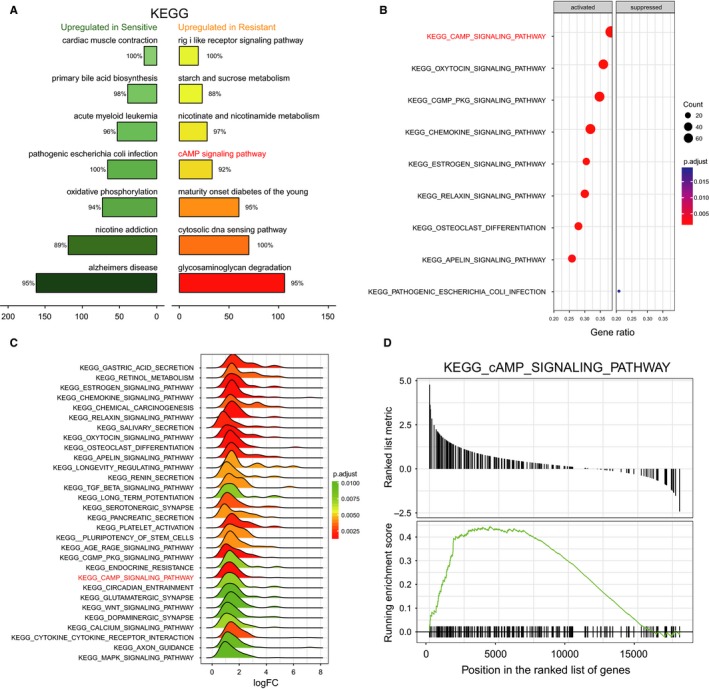
The cAMP signalling pathway was significantly activated in MTX‐resistant CRC cells (A) A rank plot of the GSEA results showing the top 9 significantly activated and inactivated signalling pathways, including the cAMP signalling pathway. *P* (adjusted) <0.05. (B) A dotplot of the GSEA results indicating that the cAMP signalling pathway was activated in MTX‐resistant CRC cells. *P* (adjusted) <0.05. (C) A ridgeplot based on the GSEA results indicating that the cAMP signalling pathway was activated. *P* (adjusted) <0.05. (D) A GSEAplot indicating that the NES value of the cAMP signalling pathway was greater than zero and that this pathway was significantly enriched in MTX‐resistant CRC cells

Multiple bioinformatics techniques were applied to investigate the differentially expressed lncRNAs and mRNAs and the significantly altered pathways in MTX‐resistant CRC cells. Initially, the top 20 up‐ and down‐regulated lncRNAs and miRNAs were screened in MTX‐resistant CRC cells using microarray analysis. Bothe KCNQ1OT1 and *PPP1R1B* were determined to be significantly up‐regulated in the MTX‐resistant CRC cells, in comparison with their levels in the MTX‐sensitive CRC cells (Figure 1A,B). Furthermore, a coexpression network of differentially expressed lncRNAs and miRNAs indicated that KCNQ1OT1 was closely associated with *PPP1R1B* (Figure 1C). Subsequently, specific miRNA targets were identified with TargetScan and miRanda, suggesting that the existence of binding sites between miR‐760 and KCNQ1OT1/*PPP1R1B* (Figure 1D). Additionally, some crucial pathways in which differentially expressed mRNAs were enriched were revealed by GSEA. The rank plot of the GSEA results shows the top 9 significantly activated or inactivated signalling pathways in MTX‐resistant CRC cells, wherein the cAMP signalling pathway was activated (Figure 2A, *P* adjusted <0.05). The dotplot and ridgeplot of the GSEA results revealed that the cAMP signalling pathway was activated (Figure 2B,C, *P* adjusted <0.05). Moreover, in the GSEAplot, a number of the differentially expressed genes were up‐regulated in the cAMP signalling pathway, indicating that the normalized enrichment score (NES) value of the cAMP signalling pathway was greater than zero (Figure 2D). Our results illustrated that the cAMP signalling pathway was significantly enriched in the MTX‐resistant CRC cells.

### 3.2 LncRNA KCNQ1OT1 was markedly overexpressed in MTX‐resistant CRC cells and tissues, and silencing of KCNQ1OT1 suppressed MTX‐resistant CRC cell survival and proliferation

**Figure 3:**
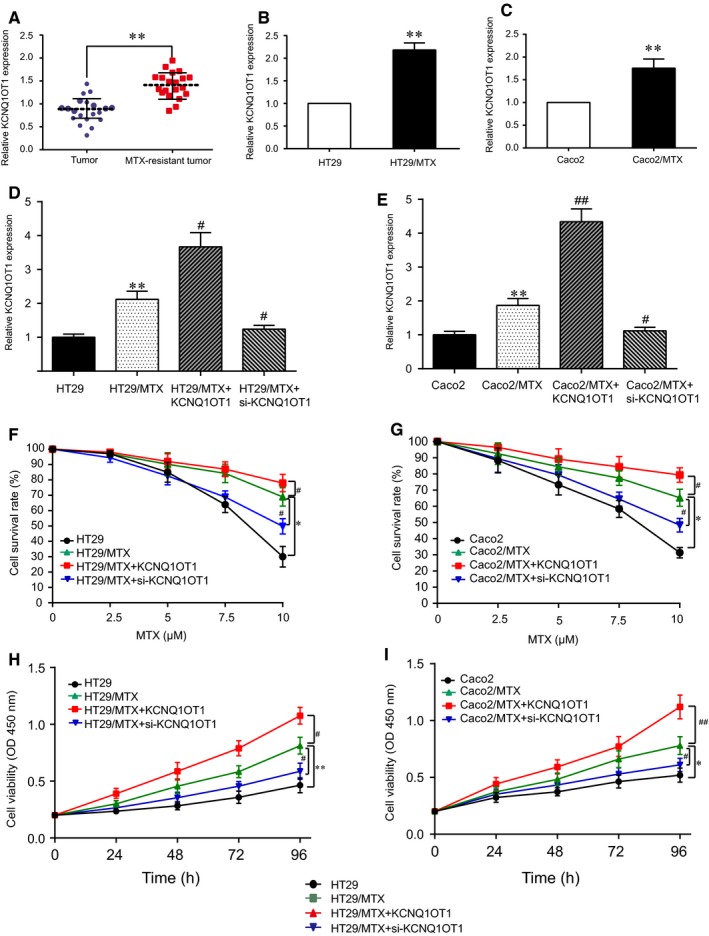
KCNQ1OT1 was overexpressed in MTX‐resistant CRC tissues and cells and influenced MTX‐resistant CRC cell survival and proliferation (A) LncRNA KCNQ1OT1 was highly expressed in MTX‐resistant CRC tissues compared with its expression in MTX‐sensitive tissues, as determined by qRT‐PCR. **P < 0.01 compared with the MTX‐sensitive tumour tissues. (B) LncRNA KCNQ1OT1 was significantly up‐regulated in MTX‐resistant HT29 cells (HT29/MTX) compared with that in HT29 cells, as determined by qRT‐PCR. *P < 0.01 compared with the HT29 cells. (C) LncRNA KCNQ1OT1 was significantly up‐regulated in MTX‐resistant Caco2 cells (Caco2/MTX) compared with the Caco2 cells, as determined by qRT‐PCR. **P < 0.01 compared with the Caco2 cells. (D) LncRNA KCNQ1OT1 expression was measured by qRT‐PCR after transfection with pcDNA3.1‐KCNQ1OT1/si‐KCNQ1OT1 in the HT29 cell line. **P < 0.01 compared with the HT29 cells, #*P* < 0.05 compared with the HT29/MTX cells. LncRNA KCNQ1OT1 expression was measured by qRT‐PCR after transfection of the Caco2 cell line. **P < 0.01 compared with that of the Caco2 cells, #*P* < 0.05 and ##*P* < 0.01 compared with the Caco2/MTX cells. (F) A cell survival assay was performed to determine the effects of MTX resistance and KCNQ1OT1 with increasing concentrations of MTX in HT29 cells. *P < 0.05 compared with the HT29 cells, #*P* < 0.05 compared with the HT29/MTX cells. (G) A cell survival assay was performed to determine the effects of MTX resistance and KCNQ1OT1 with increasing concentrations of MTX in Caco2 cells. *P < 0.05 compared with the Caco2 cells, #*P* < 0.05 compared with the Caco2/MTX cells. (H) A CCK‐8 assay was performed to confirm the effects of MTX resistance and KCNQ1OT1 on cell proliferation in HT29 cells. *P < 0.05 compared with the HT29 cells, #*P* < 0.05 compared with the HT29/MTX cells. (I) A CCK‐8 assay was performed to confirm the effects of MTX resistance and KCNQ1OT1 on cell proliferation in Caco2 cells. *P < 0.05 compared with the Caco2 cells, #*P* < 0.05 compared with the Caco2/MTX cells

qRT‐PCR analysis revealed that lncRNA KCNQ1OT1 was markedly overexpressed in MTX‐resistant CRC tissues compared with its expression in MTX‐sensitive tumour tissues (Figure 3A, *P* < 0.01). In addition, KCNQ1OT1 was also significantly up‐regulated in the MTX‐resistant cell line in comparison with the levels in the normal CRC cell line (Figure 3B,C, *P* < 0.01). In the HT29/MTX cell line, the expression of KCNQ1OT1 was higher after transfection with pcDNA 3.1‐KCNQ1OT1 plasmids, while it was reduced after transfection with si‐KCNQ1OT1 (Figure 3D); the results in the Caco2/MTX cell line were similar (Figure 3E). The cell survival assay revealed that the proliferation of the normal CRC cells decreased rapidly with increasing concentrations of MTX, while the cell survival of the MTX‐resistant cells decreased slightly. After transfection of KCNQIOT1 into the HT29/MTX cells, the reduction in survival rate gradually slowed, but transfection with si‐KCNQIOT1 suppressed the survival of the HT29/MTX cells (Figure 3F); this result was also observed in the Caco2 and Caco2/MTX cells (Figure 3G). The CCK‐8 assay further validated the influence of KCNQIOT1 on MTX‐resistant cells. These results indicated that transfection with KCNQ1OT1 considerably facilitated the cell viability of both HT29/MTX cells and Caco2/MTX cells. Conversely, silencing KCNQ1OT1 inhibited MTX‐resistant CRC cell proliferation (Figure 3H,I).

### 3.3 Silencing KCNQ1OT1 induced G0/G1 cell cycle arrest and promoted apoptosis in MTX‐resistant CRC cells

**Figure 4:**
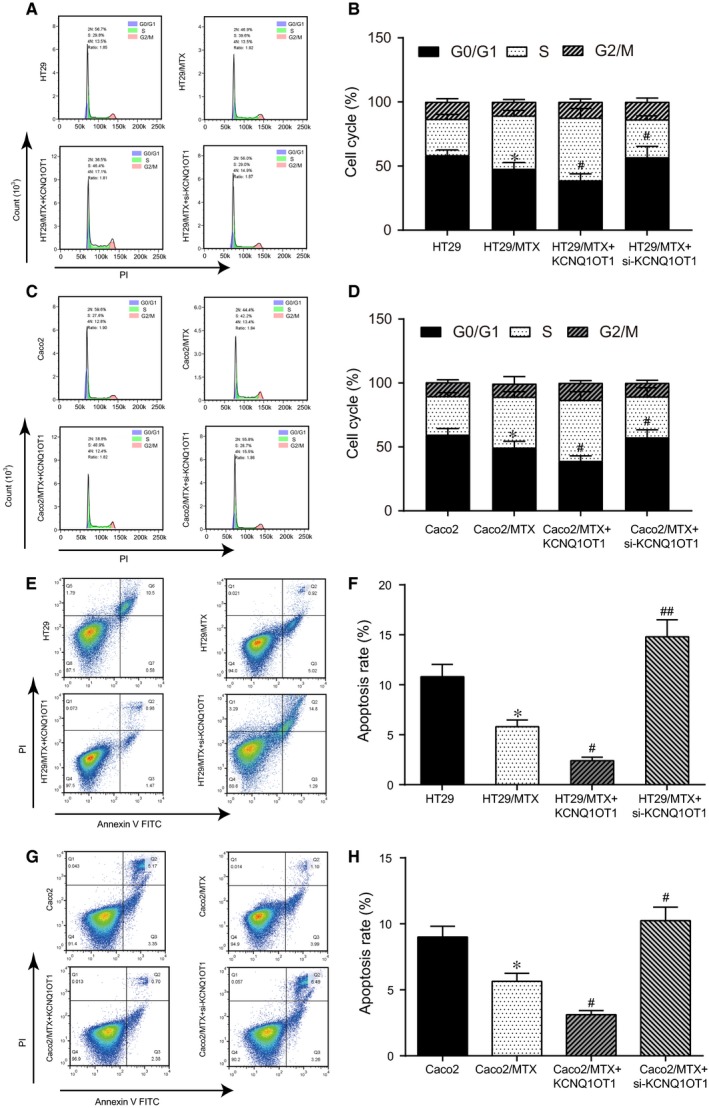
KCNQ1OT1 influenced cell cycle progression and apoptosis in MTX‐resistant CRC cells (A, B) The cell cycle stage was detected by flow cytometry in HT29 and HT29/MTX cells to explore the effect of KCNQ1OT1. *P < 0.05 compared with the HT29 cells, #*P* < 0.05 compared with the HT29/MTX cells. (C, D) The cell cycle stage was detected by flow cytometry in Caco2 and Caco2/MTX cells to explore the effect of KCNQ1OT1. *P < 0.05 compared with the Caco2 cells, #*P* < 0.05 compared with the Caco2/MTX cells. (E, F) Cell apoptosis was detected by flow cytometry in HT29 and HT29/MTX cells to explore the effect of KCNQ1OT1. *P < 0.05 compared with the HT29 cells, #*P* < 0.05 and ##*P* < 0.01 compared with the HT29/MTX cells. (G, H) Cell apoptosis was detected by flow cytometry in Caco2 and Caco2/MTX cells to explore the effect of KCNQ1OT1. *P < 0.05 compared with the Caco2 cells, #*P* < 0.05 compared with the Caco2/MTX cells

Flow cytometry assay was carried out to probe effects of KCNQ1OT1 on MTX‐resistant CRC cells towards cell cycle progression together with apoptosis. As presented in Figure 4A,B, the percentage of G0/G1 phase cells in the HT29/MTX group was significantly less than that in the HT29 group (*P* < 0.05). Following transfection with KCNQ1OT1, more HT29/MTX cells entered the S phase (*P* < 0.05), whereas the silencing of KCNQ1OT1 induced G0/G1 cell cycle arrest in the HT29/MTX cells (*P* < 0.05). KCNQ1OT1 had similar effects on the Caco2 and Caco2/MTX cells (Figure 4C,D, *P* < 0.05). Furthermore, the apoptosis rate in the HT29/MTX + KCNQ1OT1 group was lower than that in the HT29/MTX group (*P* < 0.05), whereas the apoptosis rate in the HT29/MTX+ si‐KCNQ1OT1 group was elevated (Figure 4E,F, *P* < 0.01). The results in the Caco2 and Caco2/MTX cells showed a similar trend (Figure 4G,H, *P* < 0.05). All of the results mentioned showed that KCNQ1OT1 overexpression reduced the percentage of MTX‐resistant CRC cells arrested in G0 phase and suppressed cell apoptosis, whereas the silencing of KCNQ1OT1 induced G0/G1 cell cycle arrest in MTX‐resistant CRC cells and accelerated cell apoptosis.

### 3.4 Silencing KCNQ1OT1 inhibited the proliferation of MTX‐resistant CRC cells through the sponging of miR‐760

**Figure 5:**
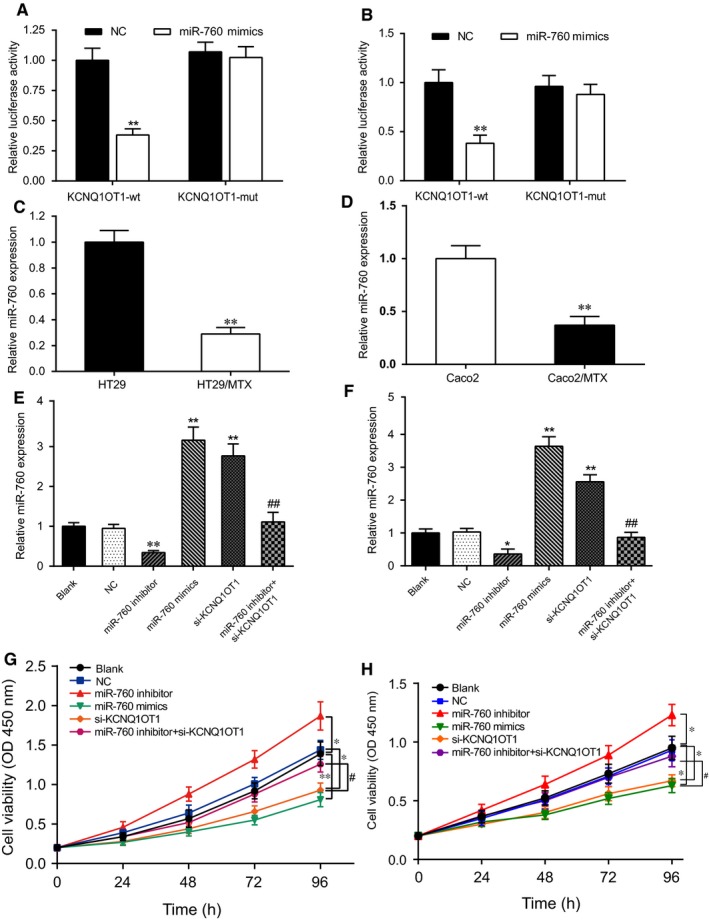
Silencing of KCNQ1OT1 influenced the proliferation of MTX‐resistant CRC cells through the sponging of miR‐760. (A) The targeted relationship between KCNQ1OT1 and miR‐760 was determined with the dual‐luciferase reporter gene assay in HT29/MTX cells under the treatment with MTX. **P < 0.01 compared with the KCNQ1OT1 +  NC group. (B) The targeted relationship between KCNQ1OT1 and miR‐760 was determined with the dual‐luciferase reporter gene assay in Caco2/MTX cells under the treatment with MTX. **P < 0.01 compared with the KCNQ1OT1 +  NC group. (C) MiR‐760 was visibly down‐regulated in HT29/MTX cells compared with its levels in HT29 cells, as determined by qRT‐PCR. **P < 0.01 compared with the HT29 cells. (D) MiR‐760 was markedly down‐regulated in Caco2/MTX cells compared with its expression in Caco2 cells, as detected by qRT‐PCR. **P < 0.01 compared with the Caco2 cells. (E) qRT‐PCR was performed to measure miR‐760 expression after transfection in HT29/MTX cells. **P < 0.01 compared with the NC group, ##*P* < 0.01 compared with the miR‐760 inhibitor group. (F) qRT‐PCR was performed to measure miR‐760 expression after transfection in Caco2/MTX cells. *P < 0.05 and **P < 0.01 compared with the NC group, ##*P* < 0.01 compared with the miR‐760 inhibitor group. (G) Cell proliferation was measured with a CCK‐8 assay to explore the effect of the KCNQ1OT1/miR‐760 axis in HT29/MTX cells. *P < 0.05 and **P < 0.01 compared with the NC group, #*P* < 0.05 compared with the miR‐760 inhibitor group. (H) Cell proliferation was measured with a CCK‐8 assay to explore the effect of the KCNQ1OT1/miR‐760 axis in Caco2/MTX cells. *P < 0.05 compared with the NC group, #*P* < 0.05 compared with the miR‐760 inhibitor group

The targeted relationship between KCNQ1OT1 and miR‐760 was confirmed with the dual‐luciferase reporter gene assay. In both HT29/MTX and Caco2/MTX cells under the treatment with MTX, the activity of the luciferase reporter gene containing the miR‐760 site was significantly reduced following transfection with miR‐760 mimics, whereas the activity of the reporter gene containing the KCNQ1OT1 mutant sequence showed no significant change, which indicated there were complementary base pairing existing between KCNQ1OT1 and miR‐760 (Figure 5A,B, *P* < 0.01). The qRT‐PCR outcomes showed that miR‐760 was noticeably down‐regulated in the MTX‐resistant CRC cells in comparison with those in the normal CRC cells (Figure 5C,D, *P* < 0.01). The expression of miR‐760 was increased after transfection with miR‐760 mimics, while it was inhibited in the miR‐760 inhibitor group in the HT29/MTX cell line (Figure S1A) and the Caco2/MTX cell line (Figure S1B). The cell survival assay demonstrated that the reduction in survival rate gradually slowed with increasing concentrations of MTX in the miR‐760 inhibitor group in both HT29/MTX cells and Caco2/MTX cells, whereas the opposite effect was observed in the miR‐760 mimic group (Figure S1C,D). Next, the upstream and downstream relationship between KCNQ1OT1 and miR‐760 was verified by qRT‐PCR, after transfection with the miR‐760 inhibitor, the expression of miR‐760 was significantly suppressed, whereas si‐KCNQ1OT1 and miR‐760 mimics considerably increased miR‐760 expression. The expression of miR‐760 in the si‐KCNQ1OT1+ miR‐760 inhibitor group was markedly elevated compared with that in the miR‐760 inhibitor group (Figure 5E,F, *P* < 0.01). On the other hand, the expression of KCNQ1OT1 was tested when cells were transfected, obviously, the expression of KCNQ1OT1 had almost no difference with NC group when both HT29/MTX cells and Caco2/MTX cells were transfected with miR‐760 mimics and inhibitor (Figure S2). All the results indicated that KCNQ1OT1 could negatively regulate the expression of miR‐760, KCNQ1OT1 was the upstream of miR‐760. The CCK‐8 assays revealed that the cell viability of the HT29/MTX cells was significantly suppressed by miR‐760 mimics and si‐KCNQ1OT1 but was elevated by the miR‐760 inhibitor. After cotransfection with si‐KCNQ1OT1 and the miR‐760 inhibitor, the viability of the HT29/MTX cells was increased compared with that of the cells in the miR‐760 inhibitor group (Figure 5G). An analogous tendency was observed in the Caco2 cells (Figure 5H). Overall, KCNQ1OT1 functioned as a sponge of miR‐760 while the silencing of KCNQ1OT1 dampened the proliferation of MTX‐resistant CRC cells via modulating miR‐760 expression.

### 3.5 Silencing of KCNQ1OT1 induced cell cycle arrest and apoptosis in MTX‐resistant CRC cells by sponging miR‐760

**Figure 6:**
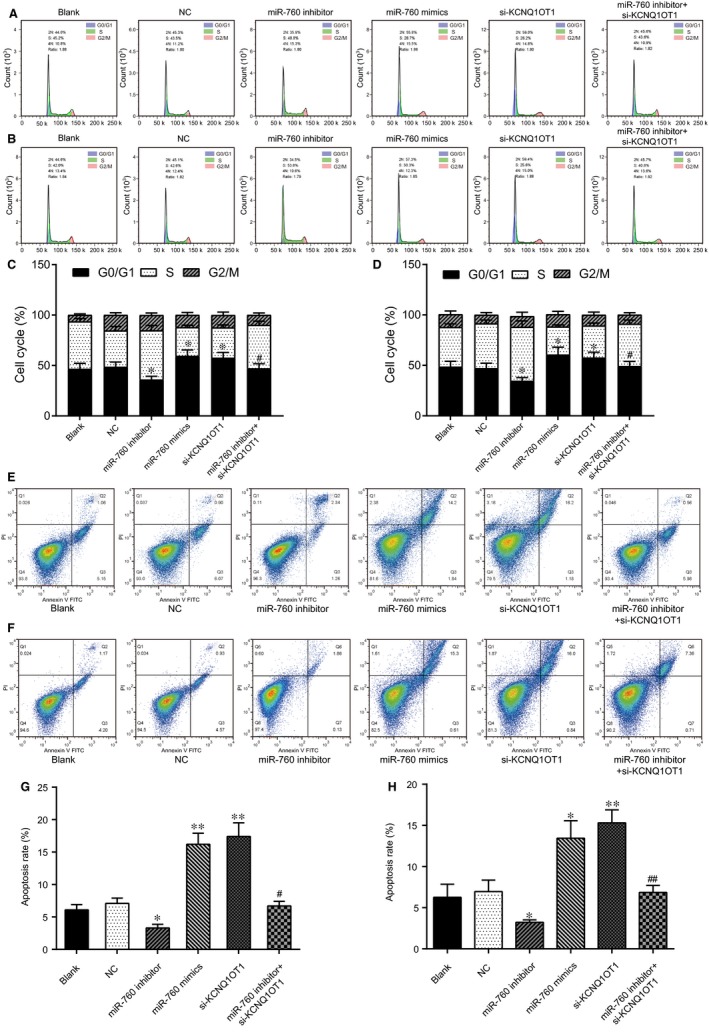
Silencing of KCNQ1OT1 influenced cell cycle progression and apoptosis in MTX‐resistant CRC cells through the sponging of miR‐760 (A, C) The cell cycle stage was determined by flow cytometry in HT29/MTX cells to explore the effect of the KCNQ1OT1/miR‐760 axis. *P < 0.05 compared with the NC group, #*P* < 0.05 compared with the miR‐760 inhibitor group. (B, D) The cell cycle stage was determined by flow cytometry in Caco2 and Caco2/MTX cells to explore the effect of the KCNQ1OT1/miR‐760 axis. *P < 0.05 compared with the NC group, #*P* < 0.05 compared with the miR‐760 inhibitor group. (E, G) Cell apoptosis was detected by flow cytometry in HT29/MTX cells to explore the effect of the KCNQ1OT1/miR‐760 axis. *P < 0.05 compared with the NC group, #*P* < 0.05 compared with the miR‐760 inhibitor group. (F, H) Cell apoptosis was detected by flow cytometry in Caco2/MTX cells to explore the effect of the KCNQ1OT1/miR‐760 axis. *P < 0.05 compared with the NC group, #*P* < 0.05 compared with the miR‐760 inhibitor group

To investigate the impacts of the KCNQ1OT1/miR‐760 axis on MTX‐resistant CRC cells, examinations of cell cycle progression and apoptosis were conducted using flow cytometry. As shown in Figure 6A,C, the number of the HT29/MTX cells arrested in the G0/G1 phase in the miR‐760 mimic and the si‐KCNQ1OT1 groups was obviously larger than that in the NC group, indicating that miR‐760 overexpression and KCNQ1OT1 knockdown both induced the arrest of G0/G1‐phase cell cycle arrest in the MTX‐resistant CRC cells. However, the miR‐760 inhibitor facilitated the entry of the HT29/MTX cells into the S phase. After cotransfected with the miR‐760 inhibitor and si‐KCNQ1OT1, the percentage of cells remaining in the G0/G1 stage was increased compared with that in the miR‐760 inhibitor group (*P* < 0.05). The influence of the KCNQ1OT1/miR‐760 axis was similar in the Caco2/MTX cells (Figure 6B,D, *P* < 0.05). Additionally, miR‐760 overexpression and KCNQ1OT1 knockdown also increased apoptosis in MTX‐resistant CRC cells, whereas the miR‐760 inhibitor suppressed MTX‐resistant CRC cell apoptosis (Figure 6E‐H). Taken together, the results indicated that the silencing of KCNQ1OT1 induced cell cycle arrest when promoting MTX‐resistant CRC cell apoptosis by targeting miR‐760.

### 3.6 MiR‐760 modulated *PPP1R1B* and downstream *CREB* and *CBP* expression in the cAMP signalling pathway

**Figure 7:**
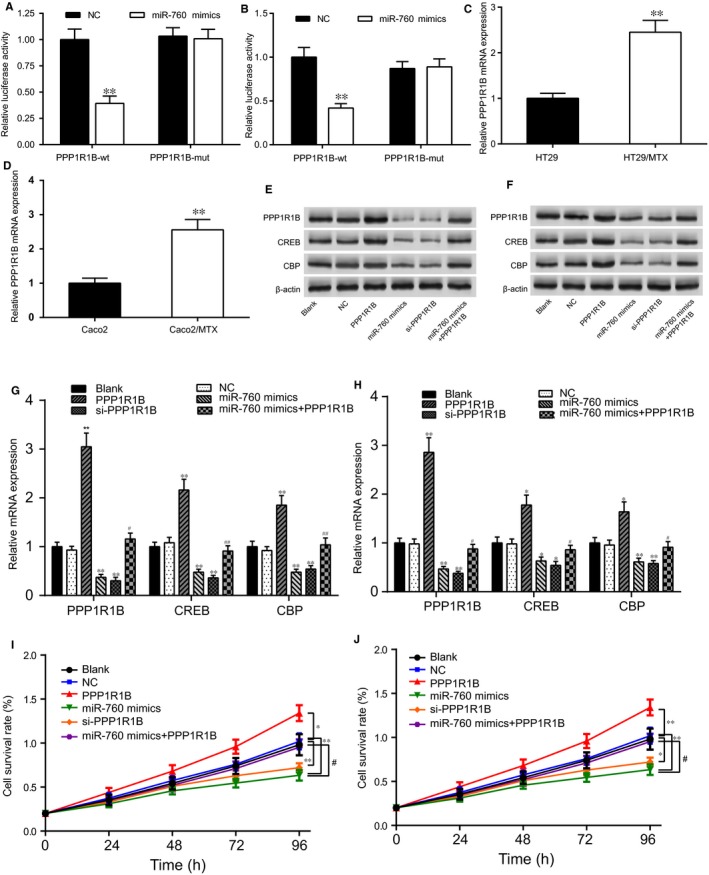
MiR‐760 modulated *PPP1R1B* expression and the downstream proteins *CREB* and *CBP* in the cAMP signalling pathway. (A) The targeted relationship between miR‐760 and *PPP1R1B* was determined by dual‐luciferase reporter assay in HT29/MTX cells under the treatment with MTX. **P < 0.01 compared with the *PPP1R1B* + NC group. (B) The targeted relationship between miR‐760 and *PPP1R1B* was determined with the dual‐luciferase reporter assay in Caco2/MTX cells under the treatment with MTX. **P < 0.01 compared with the *PPP1R1B* + NC group. (C) *PPP1R1B* was overexpressed in HT29/MTX cells, as measured by qRT‐PCR. **P < 0.01 compared with the HT29 cells. (D) *PPP1R1B* was overexpressed in Caco2/MTX cells, as measured by qRT‐PCR. **P < 0.01 compared with the HT29 cells. (E) Western blot was used to examine the influence of miR‐760 on *PPP1R1B* and the related proteins *CREB* and *CBP* in the cAMP signalling pathway in HT29/MTX cells. (F) Western blot was used to examine the influence of miR‐760 on *PPP1R1B* and the related proteins *CREB* and *CBP* in the cAMP signalling pathway in Caco2/MTX cells. (G) The expression levels of *PPP1R1B*,*CREB,* and *CBP* were measured by qRT‐PCR to explore the effect of the *PPP1R1B*/miR‐760 axis in HT29/MTX cells. **P < 0.01 compared with the NC group, #*P* < 0.05 and ##*P* < 0.01 compared with the *PPP1R1B* group. (H) The expression levels of *PPP1R1B*,*CREB* and *CBP* were measured by qRT‐PCR to explore the effect of the *PPP1R1B*/miR‐760 axis in Caco2/MTX cells. *P < 0.05 and **P < 0.01 compared with the NC group, #*P* < 0.05 compared with the *PPP1R1B* group. (I) Cell viability was determined with a CCK‐8 assay to explore the effect of the *PPP1R1B*/miR‐760 axis in HT29/MTX cells. *P < 0.05 and **P < 0.01 compared with the NC group, #*P* < 0.05 compared with the *PPP1R1B* group. (H) Cell viability was determined with a CCK‐8 assay to explore the effect of the *PPP1R1B*/miR‐760 axis in Caco2/MTX cells. *P < 0.05 and **P < 0.01 compared with the NC group, #*P* < 0.05 compared with the *PPP1R1B* group

The targeted relationship between *PPP1R1B* and miR‐760 was verified with the dual‐luciferase reporter gene assay. Cotransfection with the miR‐760 expression vector was found to decrease wild‐type *PPP1R1B* 3′‐UTR reporter activity compared with the activity after transfection with empty vector controls, whereas no effect was observed on mutant *PPP1R1B* 3′‐UTR reporter activity in either HT29/MTX cells or Caco2/MTX cells under the treatment with MTX (Figure 7A,B, *P* < 0.01). The qRT‐PCR results revealed that *PPP1R1B* was significantly up‐regulated in the MTX‐resistant CRC cells compared with the levels in the normal CRC cells (Figure 7C,D, *P* < 0.01). *PPP1R1B* mRNA expression was increased after transfection with pcDNA3.1‐*PPP1R1B* but was reduced after transfection with si‐*PPP1R1B* in the HT29/MTX (Figure S1E) and Caco2/MTX cell lines (Figure S1F). The results of the cell survival assay indicated that the survival rate was gradually reduced in the HT29/MTX + *PPP1R1B* group, whereas the opposite effect was observed in the HT29/MTX + si‐*PPP1R1B* group (Figure S1G). In addition, the effects of *PPP1R1B* on the resistance to MTX were similar in the Caco2/MTX cells (Figure S1H). After transfection with si‐*PPP1R1B* or miR‐760 mimics, the mRNA and protein expression of *PPP1R1B* were significantly suppressed. Compared with the levels in the *PPP1R1B* group, the PPP1R1B mRNA and protein levels in the miR‐760 mimics + *PPP1R1B* group were clearly repressed (Figure 7E‐H). Thereafter, Western blot and qRT‐PCR were utilized to reveal the influence of the miR‐760/*PPP1R1B* axis on the *PPP1R1B*‐mediated downstream expression of *CREB* and *CBP* in the cAMP signalling pathway. As shown in Figure 7E‐H, The mRNA and protein expression of *CREB* and *CBP* were considerably repressed by si‐*PPP1R1B* and miR‐760 mimics and dramatically up‐regulated by *PPP1R1B* cDNA. The expression levels of CREB and CBP mRNA and protein in the *PPP1R1B* + miR‐760 mimic group were significantly suppressed when comparing with *PPP1R1B* group (Figure 7E‐H, *P < 0.05, **P < 0.01). Overall, miR‐760 regulated the expression of *PPP1R1B* and its downstream proteins *CREB* and *CBP* in the cAMP signalling pathway.

### 3.7 MiR‐760 inhibited the proliferation of MTX‐resistant CRC cells together with inducing cell cycle arrest and apoptosis by regulating *PPP1R1B*

**Figure 8:**
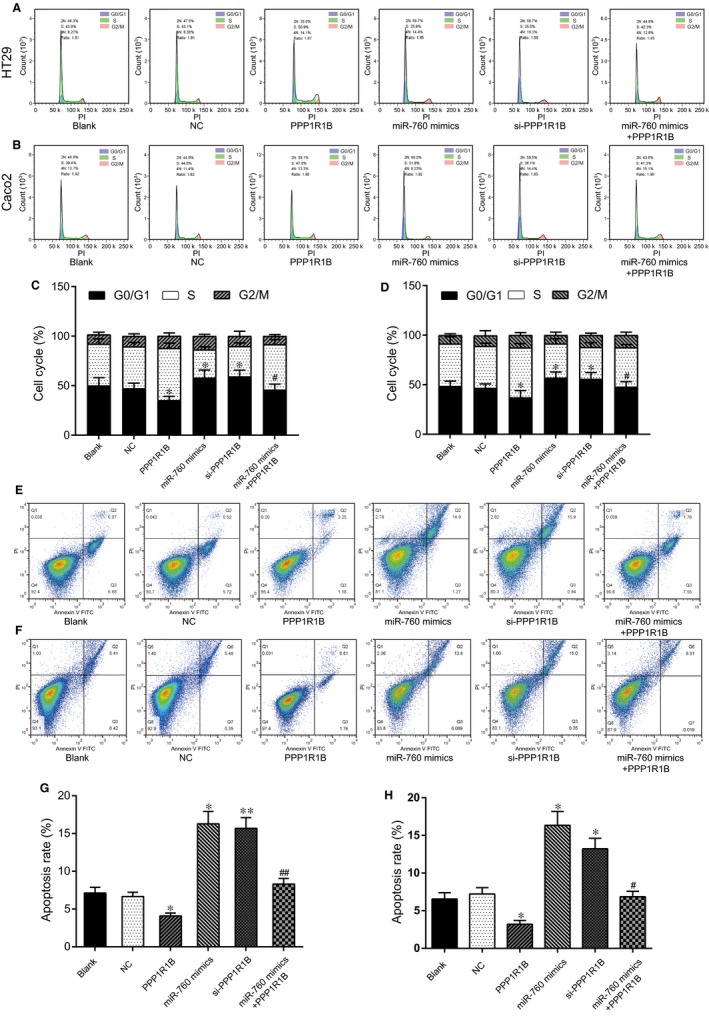
MiR‐760 induced cell cycle arrest and apoptosis by regulating *PPP1R1B* in MTX‐resistant CRC cells. (A, C) The cell cycle stage was detected by flow cytometry in HT29/MTX cells to explore the effect of the miR‐760/*PPP1R1B* axis. *P < 0.05 and **P < 0.01 compared with the NC group, #*P* < 0.05 compared with the *PPP1R1B* group. (B, D) The cell cycle stage was determined by flow cytometry in Caco2/MTX cells to explore the effect of the miR‐760/*PPP1R1B* axis. *P < 0.05 compared with the NC group, #*P* < 0.05 compared with the *PPP1R1B* group. (E, G) Cell apoptosis was detected by flow cytometry in HT29/MTX cells to explore the effect of the miR‐760/*PPP1R1B* axis. *P < 0.05 and **P < 0.01 compared with the NC group, ##*P* < 0.01 compared with the *PPP1R1B* group. (F, H) Cell apoptosis was detected by flow cytometry in Caco2/MTX cells to explore the effect of the miR‐760/*PPP1R1B* axis. *P < 0.05 compared with the NC group, #*P* < 0.05 compared with the *PPP1R1B* group

The cell viability of the HT29/MTX cells was significantly suppressed by si‐*PPP1R1B* and miR‐760 mimics and increased by pcDNA3.1‐*PPP1R1B*. In addition, the effects of *PPP1R1B* on cell viability were reversed by miR‐760 mimics (Figure 7I). The results for the Caco2/MTX cells showed a similar trend (Figure 7J). The impact of the miR‐760/*PPP1R1B* axis on cell cycle progression and apoptosis in the MTX‐resistant cells was examined using flow cytometry. The percentage of MTX‐resistant CRC cells arrested in the G0 phase in the miR‐760 mimic group and the si‐*PPP1R1B* group was notably higher than that in the NC group, suggesting that miR‐760 overexpression and *PPP1R1B* knockdown induce G0 cell cycle arrest in MTX‐resistant CRC cells. Nonetheless, *PPP1R1B* overexpression facilitated the entry of the MTX‐resistant CRC cells into the S phase. After cotransfection with miR‐760 mimics and *PPP1R1B*, the percentage of cells remaining in the G0 stage was increased (Figure 8A‐D). Additionally, miR‐760 overexpression and *PPP1R1B* knockdown increased apoptosis in the MTX‐resistant CRC cells, whereas up‐regulation of *PPP1R1B* expression suppressed MTX‐resistant CRC cell apoptosis (Figure 8E‐H). MiR‐760 accelerated MTX‐resistant CRC cell apoptosis by regulating *PPP1R1B*.

### 3.8 Silencing of KCNQ1OT1 impeded MTX‐resistant CRC tumour growth in nude mice

**Figure 9:**
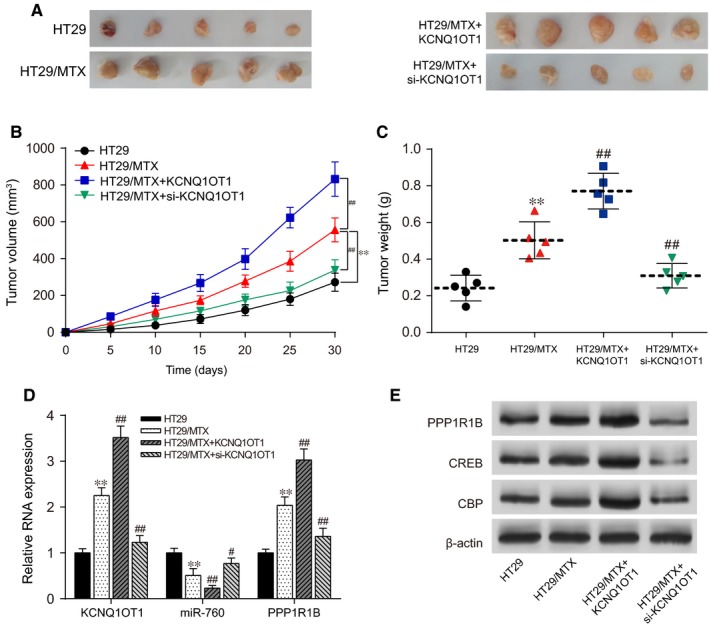
Silencing of KCNQ1OT1 impeded MTX‐resistant CRC tumour growth in vivo. (A) Images of tumour tissues. (B) The tumour volumes of nude mice injected with HT29/MTX cells in the KCNQ1OT1 group were significantly greater than those in the HT29/MTX group, whereas those in the si‐KCNQ1OT1 group were relatively smaller. (C) The tumour masses of nude mice injected with HT29/MTX cells in the KCNQ1OT1 group were significantly heavier than those in the HT29/MTX group, whereas those in the si‐KCNQ1OT1 group were relatively lighter. (D) The influence of KCNQ1OT1 on miR‐760 and *PPP1R1B* expression, as determined by qRT‐PCR. (E) The influence of KCNQ1OT1 on *PPP1R1B* expression and the expression of the related proteins CREB and CBP in the cAMP signalling pathway, as determined by Western blot. *P < 0.05 and **P < 0.01 compared with the HT29 group, #*P* < 0.05 and ##*P* < 0.01 compared with the HT29/MTX group

The impact of KCNQ1OT1 on MTX‐resistant CRC tumour growth and related proteins in the cAMP signalling pathway was examined with a tumour xenograft assay. As demonstrated in Figure 9A‐C, the tumour mass volume and the tumour mass weight of the nude mice injected with HT29/MTX cells were much larger than those of mice injected with HT29 cells (*P* < 0.01). Additionally, the tumour mass of the nude mice in the HT29/MTX + KCNQ1OT1 group was greater than that of the HT29 group and the HT29/MTX group, whereas the tumour mass in the HT29/MTX + si‐KCNQ1OT1 group was relatively smaller than that of the HT29/MTX group. These results also suggested that silencing KCNQ1OT1 might restrain tumour growth in HT29/MTX mice. In addition, the results of qRT‐PCR represented that the expression level of KCNQ1OT1 and *PPP1R1B* in the HT29/MTX mice was much higher than in the HT29 mice, but the expression of miR‐760 presented an opposite trend. Moreover, pcDNA3.1‐KCNQ1OT1 considerably increased the expression of KCNQ1OT1 and *PPP1R1B* in the HT29/MTX + KCNQ1OT1 group, whereas si‐KCNQ1OT1 significantly increased the miR‐760 expression (Figure 9D). Finally, Western blot analysis revealed the influence of KCNQ1OT1 on protein expression in the cAMP signalling pathway. As demonstrated in Figure 9E, the protein expression of *PPP1R1B1* and its downstream proteins CREB and CBP were much higher in the HT29/MTX group than in the HT29 group. The protein expression of PPP1R1B1, CREB, and CBP was significantly enhanced by pcDNA3.1‐KCNQ1OT1 but was inhibited by si‐KCNQ1OT1.

## 4 DISCUSSION

Our experimental results and statistical analyses determined that lncRNA KCNQ1OT1 and *PPP1R1B* mRNA were aberrantly up‐regulated in MTX‐resistant CRC tumour tissues and cells. However, knockdown of KCNQ1OT1 increased the cell chemosensitivity towards MTX, reduced MTX‐resistant HT29/MTX cell viability and propagation when it also resulted in cell cycle arrest and apoptosis. Regarding the detailed mechanism, KCNQ1OT1 acted as a sponge of miR‐760, which targeted *PPP1R1B*. Under MTX treatment, low expression of miR‐760 activated the cAMP signalling pathway by regulating the genes *CREB* and *CBP*.

Methotrexate has a long history of serving as a cytotoxic drug in clinical treatment. Although it is commonly applied in the treatment of different cancers, the frequent occurrence of MTX resistance in tumours in such cancers as CRC,^7^ breast cancer,^19^ acute nonlymphocytic leukaemia,^20^ and gestational trophoblastic neoplasia^21^ brings severe challenges to therapy. Aiming at exploring the mechanism of MTX resistance in CRC, we found a closely interrelated regulating chain.

Knockdown of KCNQ1OT1 increased the chemosensitivity towards MTX. The relationship between KCNQ1OT1 and drug resistance had been reported in other chemotherapeutic drug resistance studies. KCNQ1OT1 acted as a potential oncogene that inhibited the malignancy and chemoresistance of lung adenocarcinoma cells,^22^ and Huiqin Hu et al also indicated that KCNQ1OT1 modulated oxaliplatin resistance in hepatocellular carcinoma through the miR‐7‐5p/ABCC1 axis.^23^ Furthermore, Zhang et al reported that knockdown of KCNQ1OT1 inhibited tongue squamous cell carcinoma cell proliferation and cisplatin resistance.^24^

Dysregulation of miR‐760 has been found in tumour tissues. On the one hand, overexpression of miR‐760 accelerates the proliferation of ovarian cancer cells.^25^ On the other hand, miR‐760 suppresses breast cancer cell multiplication and metastasis^26^ and is down‐regulated in the medulla osmium and primary tumour of terminal gastric cancer patients.^27^ In terms of CRC, research has shown that miR‐760 is closely connected with the processes of proliferation and invasion in CRC cells through the targeting of downstream mRNAs through related signalling pathways^16^; it is also down‐regulated in CRC cells and might serve as a prognostic biomarker.^28^ Moreover, miR‐760 mediates chemoresistance in cancer.^29^ Consistent with previous findings, miR‐760 acted as a tumour suppressor in CRC and that low miR‐760 expression was shown in our study, and it may be involved in cancer cells’ resistance to oncology drugs, including MTX.

*PPP1R1B*, together with its downstream proteins, is overexpressed in diverse adenocarcinomas, including colon cancer. Experiments and statistical analyses have been implemented to verify this argument and have discovered that *PPP1R1B* promotes chemoresistance by regulating pro‐oncogenic signal transduction pathways.^17^ MiRNAs down‐regulate gene expression through sequence‐specific interactions with mRNA targets.^13^ In our study, the results indicated that miR‐760 could target *PPP1R1B* by interacting with highly conserved sequences in the 3′UTR, supplementing the results of previous studies.

However, this study has several limitations. For example, due to the difficulties in conducting clinical experiments, we did not clinically verify our conclusions. Furthermore, there were several other enriched signalling pathways in the GSEA results, so additional signalling pathways related to MTX resistance should be explored with enrichment analysis. In addition, the role of the KCNQ1OT1/miR‐760/*PPP1R1B* axis should be investigated in other mechanisms of cancer cells resistance to chemotherapeutics.

In conclusion, the present work clarifies the regulatory mechanism of the KCNQ1OT1/miR‐760/*PPP1R1B* axis in MTX resistance in CRC involving the cAMP signalling pathway. KCNQ1OT1 is up‐regulated in MTX‐resistant CRC tumour tissues and acts as the sponge of miR‐760, thus promoting the expression of the target gene *PPP1R1B* by activating the cAMP signalling pathway. Overall, overexpression of lncRNA KCNQ1OT1 increases the risk of chemoresistance to the anticancer drug MTX, leading to poor therapeutic efficacy. This study has found a potential role for KCNQ1OT1 as a new therapeutic target and prognostic factor, thus providing a possible future therapy against MTX resistance in CRC.

## CONFLICT OF INTEREST

No conflict of interest exits in the submission of this manuscript.

## AUTHOR CONTRIBUTION

Substantial contribution to the conception and design of the work: Di Xian and Yu Zhao; Analysis and interpretation of the data: Di Xian and Yu Zhao; Drafting the manuscript: Di Xian and Yu Zhao; Revising the work critically for important intellectual content: Di Xian and Yu Zhao; Collecting of grants: Yu Zhao. Final approval of the work: all authors.
