# Supplementary figures and images for: LncRNA KCNQ1OT1 enhanced the methotrexate resistance of colorectal cancer cells by regulating miR‐760/PPP1R1B via the cAMP signalling pathway
Source: J Cell Mol Med. 2019 Apr 17;23(6):3808–23. doi: 10.1111/jcmm.14071 (PMC6533496; doi:10.1111/jcmm.14071)

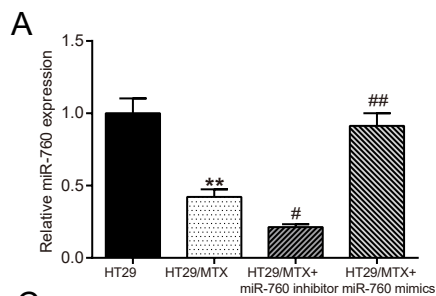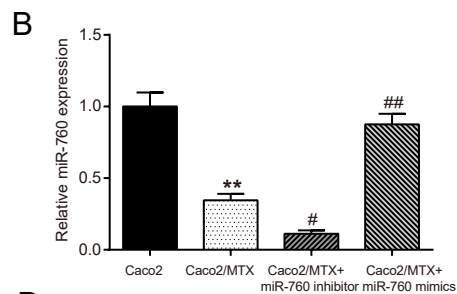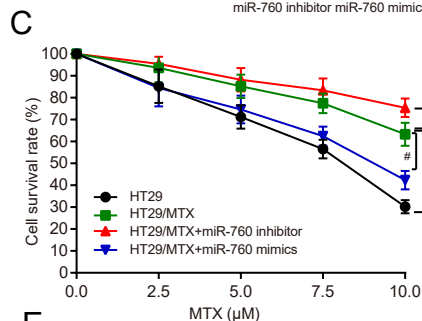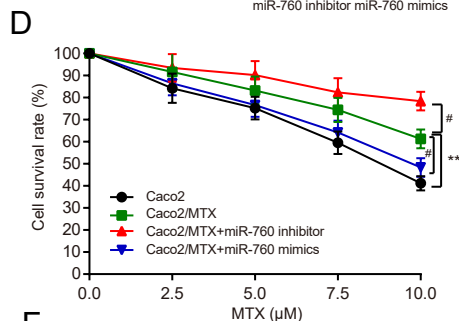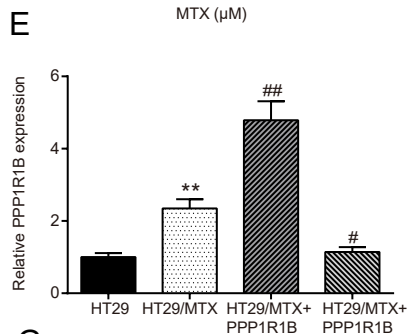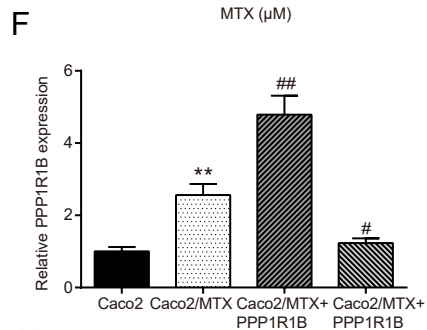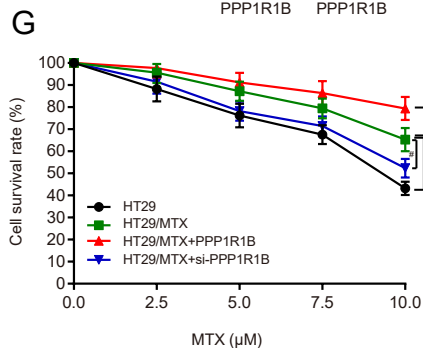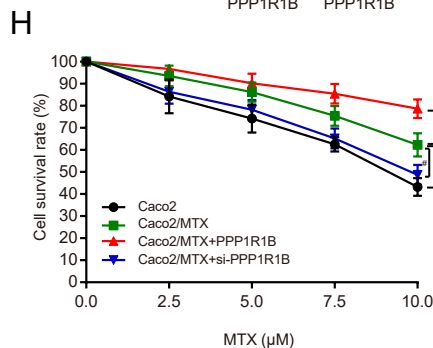

Supplement: Supplementary file 1 [file JCMM-23-3808-s001.pdf]

**A**

HT29/MTX

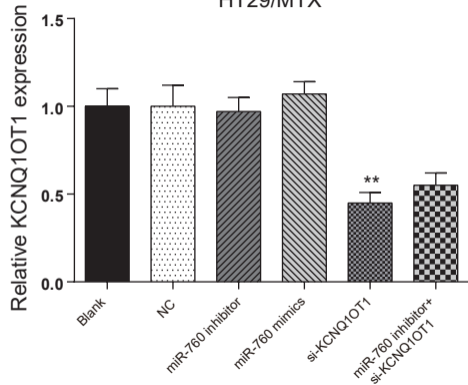**B**

Caco2/MTX

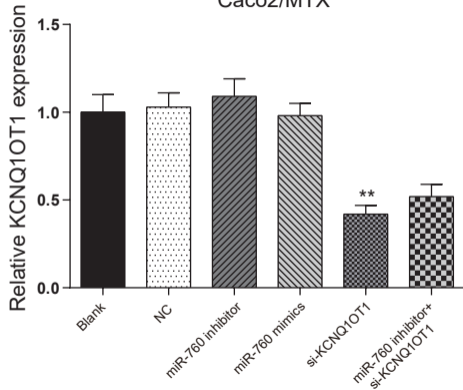

Supplement: Supplementary file 2 [file JCMM-23-3808-s002.pdf]
